# Mapping carcass and meat quality QTL on *Sus Scrofa *chromosome 2 in commercial finishing pigs

**DOI:** 10.1186/1297-9686-41-4

**Published:** 2009-01-05

**Authors:** Henri CM Heuven, Rik HJ van Wijk, Bert Dibbits, Tony A van Kampen, Egbert F Knol, Henk Bovenhuis

**Affiliations:** 1 Animal Breeding and Genomics Centre, Wageningen University, PO Box 338, 6700 AH Wageningen, The Netherlands; 2 Clinical Sciences of Companion Animals, Faculty of Veterinary medicine, Utrecht University, PO Box 80163, 3508 TD Utrecht, The Netherlands; 3 IPG-Institute for Pig Genetics B.V., PO Box 43, 6640AA Beuningen, The Netherlands

## Abstract

Quantitative trait loci (QTL) affecting carcass and meat quality located on SSC2 were identified using variance component methods. A large number of traits involved in meat and carcass quality was detected in a commercial crossbred population: 1855 pigs sired by 17 boars from a synthetic line, which where homozygous (A/A) for *IGF2*. Using combined linkage and linkage disequilibrium mapping (LDLA), several QTL significantly affecting loin muscle mass, ham weight and ham muscles (outer ham and knuckle ham) and meat quality traits, such as Minolta-L* and -b*, ultimate pH and Japanese colour score were detected. These results agreed well with previous QTL-studies involving SSC2. Since our study is carried out on crossbreds, different QTL may be segregating in the parental lines. To address this question, we compared models with a single QTL-variance component with models allowing for separate sire and dam QTL-variance components. The same QTL were identified using a single QTL variance component model compared to a model allowing for separate variances with minor differences with respect to QTL location. However, the variance component method made it possible to detect QTL segregating in the paternal line (*e.g*. HAMB), the maternal lines (*e.g*. Ham) or in both (*e.g*. pHu). Combining association and linkage information among haplotypes improved slightly the significance of the QTL compared to an analysis using linkage information only.

## Introduction

Pig breeding programs aim at improving pigs for economically important traits. Carcass quality has been successfully improved in most selection programs because phenotypes are easy to obtain on live animals via ultrasonically measurements of backfat and because these traits show a relatively high heritability. However, although breeding for meat quality has received much attention over the past two decades, it has not been the priority in most selection programs [1-4] because meat quality traits can only be measured on the relatives of selection candidates and late in life. Successful improvement of meat quality may be possible by combining molecular information and traditional measurements because marker data can be obtained on all animals at an early age [5].

Molecular information, *i.e*. genes and QTL, has rapidly become available via genome scans of experimental crossbred populations (see review by Bidanel and Rothschild [6] and PigQTLdb [7]). In many cases, favourable QTL cannot be exploited due to the poor performance of these exotic breeds with respect to commercially relevant traits. However, the number of QTL studies using commercial populations is increasing [8-22]. Identification of QTL using commercial lines requires a large number of families because fewer heterozygous founders are expected especially for traits under selection such as carcass quality traits.

Most of the studies mentioned above use 'paternal half sib regression' as the statistical method to associate genotypes with phenotypes, which models the segregation of paternal QTL [23]. Variance component methods, based on the theory developed by Fernando and Grossman [24], are currently becoming the method of choice in association studies because they allow for much greater flexibility in the modelling of QTL in arbitrary pedigrees while adjusting simultaneously for systematic environmental effects [13,25]. A preliminary analysis using eight half-sib families, detected putative QTL on SSC2 [15]. Based on these results, nine additional families were genotyped and analysed to increase the marker density in regions of interest. The goal of this paper is to map QTL affecting meat and carcass quality of commercial finishers and located on SSC2 using variance component methods.

## Methods

### Population and phenotypes

The 1855 commercial finishers were a cross product of 17 boars of a synthetic sire line (Large White/Pietrain, TOPIGS, The Netherlands) and 239 unregistered hybrid sows. The piglets were born during a two-month period in 2002. Piglets were individually tagged at birth and males were castrated three to five days after farrowing. Pigs were weaned on average at 17 days of age and raised till an average weight of 22.7 kg before being moved to the finishing barns. Diets comprised commercial available feeds and free access to water.

Pigs were loaded in three batches per compartment at an average weight of 118 kg live weight and kept overnight in a lairage at the slaughterhouse. The average age (**AGE**) of each batch was 164, 172 and 185 days, respectively. During a 70-day period, pigs were slaughtered on 17 different days. Measurements on the carcass were recorded on one half of the carcass. Backfat (**BF**) and loin depth (**LD**) were measured at the 10^th ^rib using the Hennessy grading probe HGP Systems Ltd, Auckland NZ). Lean percentage (**PLEAN**) was calculated as: PLEAN = 58.86 - (0.61 × BF) + (0.12 × LD). Cold carcass weight (**CCW**) was recorded after temperature equalization. Primal cuts of ham (**HAM**) and loin (**LOIN**) were weighed and further dissected into boneless subprimals and individual muscles. Skin and fat were removed from hams removed and four subprimals were weighted: inside ham (**IHAM**), outer ham (**OHAM**), knuckle ham (**KHAM**) and the lite butt ham (**LBHAM**,*i.e*. part of the *gluteus medius *muscle). Together they summed to boneless ham muscle weight (**BHAM**). Loins were processed to a boneless loin without the fat cover (**DLOIN**).

Meat quality measurements were taken both on the loin and the ham. Ultimate pH (**pHu**) was measured in the boneless loin 24–28 h post mortem. Loin Minolta L*, a* and b* (**LOINL**, **LOINA **and **LOINB**) were taken on the fresh cut surface of a 2.5-cm chop removed from the sirloin end using a Minolta CR 300 (Minolta, Osaka, Japan). The same chop was used for a subjective colour score (score 1 to 6, with 1 = pale and 6 = very dark) using the Japanese colour scale (**JCScut**). The side view of the loin was also scored using this scale (**JCSrib**). A subjective marbling score (**LMARB**; 1 to 5, with 1 = devoid and 5 = overly abundant) was given to the chop based on marbling standards of the National Pork Producers Council [26]. Cores were taken from a second 2.5-cm chop using a 25-mm coring device to determine drip loss percentage (**DRIP**). Samples were weighed and put in pre-weighed tubes and stored in a cooler. After 24 h samples were reweighed and drip loss was calculated [27]. Purge loss (**PURGE**, %) was determined by weighing a 7.5- to 10-cm piece of the remainder of the boneless loin, cooling it for 5 days in plastic bags and reweighing. Subjective firmness scores (**FIRM**; 1 to 3, 1 = soft and exudative and 3 = firm) were evaluated using NPPC standards [28].

Meat quality measurements taken on the ham included Minolta L*, a* and b* values on the fresh cut surface of the inside ham muscle (**HAML**, **HAMA **and **HAMB**). A subjective marbling score (**HMARB**; 1 to 4; 1 = devoid and 4 = abundant) was assigned to the outside ham muscle. General statistics regarding the data is given in van Wijk *et al*. [29].

### Genotyping and linkage map

DNA was extracted from ear or loin tissue samples using the Puregene^® ^DNA Isolation kit (D-70KA, Gentra Systems, Minneapolis, USA). Isolated DNA was tested on 1.2% agarose gel for quality and adjusted in NaCl-Tris-EDTA (STE) buffer to a final concentration of 15 ng/μL. Genotyping was performed in two batches. First eight half-sib families were typed for 10 microsatellite markers on SSC2 [15]. Next, nine additional families were genotyped for eight markers (out of the 10 markers previously used). Subsequently 16 microsatellite markers were added to fine-map regions on SSC2 based on preliminary analyses. All boars were genotyped for *IGF2 *and they were homozygous (A/A). The markers included in the statistical analysis are shown in Table 1. Genotypes were scored in duplicate and checked against pedigree information. Crimap 2.4 [30] was used to construct a sex-average linkage map. Resulting recombination fractions/cM distances were used in Simwalk version 2.89 [31] to reconstruct haplotypes, which were used in QTL analyses. Distances calculated with the Haldane linkage function were used in QTL analyses while distances calculated with the Kosambi linkage function are reported for comparison with QTL locations given in the literature [7].

**Table 1:** Linkage maps for SSC2 compared to the USDA-MARC map using the Kosambi mapping function and average distances among markers

Marker	Own data Morgan	USDA Morgan
SwC9^1^	0.00	0.00
Sw2623	0.10	0.09
SwR1910	0.24	0.24
SwR783	0.28	0.23
S0141	0.35	0.30
Sw240	0.47	0.41
Sw2513	0.51	0.41
Sw1201	0.58	0.44
Sw1686	0.60	0.45
Sw2167	0.70	0.56
Sw1655	0.75	0.63
Sw2193	0.76	0.63
ADM	0.80	0.63
SCAMP	0.82	0.72
Sw766	0.86	0.74
S0010	0.90	0.77
Sw1695	0.95	0.80
S0370	1.01	0.84
swR2157	1.05	0.88
Sw1879	1.15	1.01
Sw2514	1.21	1.03
SwR345	1.32	1.13
SwR308	1.47	1.27
S0036	1.51	1.31
		
avg. dist.	0.07	0.06

^1 ^on the USDA-map SwC9 is at 0.006 Morgan

### Statistical analysis

QTL were mapped based on a combined linkage disequilibrium and segregation analysis using the variance component method because this method uses both the segregation from the sires and the dams, uses linkage disequilibrium among haplotypes in the founders, allows for simultaneously estimation of polygenic-, QTL-, litter- and fixed-effects and allows for complex pedigrees (half- and full-sib structure). Identity by descent (IBD) probabilities of haplotypes, using reconstructed haplotypes, were calculated using the LDLA package [32], which is based on the theory developed by Meuwissen and Goddard [33]. IBD probability matrices were calculated at the midpoint of each bracket of flanking markers. The likelihood at each evaluation point was determined using ASREML [34]. For comparison reasons, models were also fitted ignoring the linkage disequilibrium (LA-only).

Phenotypes were analysed according to the following model. Since the pedigree of the sows was not available a sire-dam model was used (one component model):

(1) **y **= **Xb **+ **Zs **+ **Sc **+ **Wv **+ **e**,

where **y **is a vector containing phenotypic values, **b **is a vector containing non-genetic effects, **s **is a vector containing polygenic sire effects, **c **is a vector containing common litter and dam effects, **v **is a vector containing haplotype effects due to a putative QTL and **e **contains the residual effects. Non-genetic effects considered were a barn-group-batch, and sex as class variables and 'cold carcass weight' and 'days in the finishing barn' as linear covariables. The random effects of **s**, **c**, **v **and **e **were assumed to be normally distributed with zero mean and variances **Aσ^2^_s_**, **Iσ^2^_c_**, **G_p_σ^2^_v _**and **Iσ^2^_e_**, respectively where **A **is the genetic relationship matrix among the sires including five generations of known pedigree, **G**_**p **_is the IBD matrix among the haplotypes at evaluation point **p **and **I **is an identity matrix. **X**, **Z**, **S **and **W **are incidence matrices relating effects to phenotypes.

To relax the assumption of equal variance among the paternal and maternal haplotypes in model 1 the following model (2) was applied:

(2) **y **= **Xb **+ **Zu **+ **Sc **+ **W**_**s**_**v**_**s **_+**W**_**d**_**v**_**d **_+ **e**.

In model 2, a separate variance component is fitted for the paternal (**v**_**s**_) and maternal (**v**_**d**_) haplotypes (two-component model). Since the sires and the anonymous hybrid dams originated from different populations different QTL-alleles may be segregating at the QTL.

### Test statistic and significance threshold

To test the hypothesis of the presence of a QTL (H_1_) versus no QTL (H_0_) the likelihood ratio test (LRT) was applied. The LRT statistic at each midpoint between adjacent markers was calculated as twice the difference between the log likelihood of model 1 (or 2) minus the log likelihood of a model without a QTL effect. The test statistic plotted along the chromosome gave a LRT-profile. Given this profile, thresholds were calculated which take multiple testing across the chromosome into account using the method described by Piepho [35]. Since different likelihood profiles were obtained for each model and trait specific threshold values were obtained for each combination, significance was tested using this specific threshold.

## Results and discussion

### Map construction

Genetic linkage maps are presented in Table 1. The order of the markers and the distance among markers is in close agreement with the USDA-MARC.2 genetic linkage map [36] except for marker pair SWR1910-SWR783, which is reversed and separated by 14 cM instead of 1 cM. The average distance among the markers is 6 cM.

### QTL

The LRT statistics for traits that exceeded a Piepho-corrected threshold value of 0.05 and the position of their maximum value are given in Table 2. Depending on the trait analysed, the 0.05 threshold obtained corresponded with a nominal p-value of around 0.005. Few false positive QTL will be found at the expense of false negatives using these strict thresholds. Use of a commercial population that has been under selection for several decades might be another reason for the number of QTL observed in this study.

**Table 2:** LRT statistics of traits with a significant QTL-effect and most likely QTL location

Model:	One component model (1)				Two component model (2)			
	LDLA analysis		LA-only analysis		LDLA analysis		LA-only analysis	
Trait	LRT	cM	LRT	cM	LRT	cM	LRT	cM
HAML	9.16 *,1	26	9.98 *	26	12.72 *	17	14.76 *	26
HAMB	6.98 ^ns^	140	9.60 *	149	14.74 *	149	11.36 *	149
pHu	8.90 *	65	11.14 *	65	10.14 *	65	11.38 ^ns^	65
JCSrib	10.97 ***	140	10.46 *	140	12.21 ***	140	11.82 *	140
								
HAM	8.39 *	103	2.91 ^ns^	5	8.75 *	103	4.07 ^ns^	5
OHAM	10.48 **	103	5.42 ^ns^	103	8.48 *	103	5.44 ^ns^	103
KHAM	8.04 *	140	6.82 ^ns^	149	10.38 *	149	6.86 ^ns^	140
DLOIN	14.71 ***	73	10.94 *	73	13.08 ***	73	10.98 ^ns^	73

^1 ^ns means not significant, * < 0.5, ** < .01 and *** < .005

**Figure 1:**
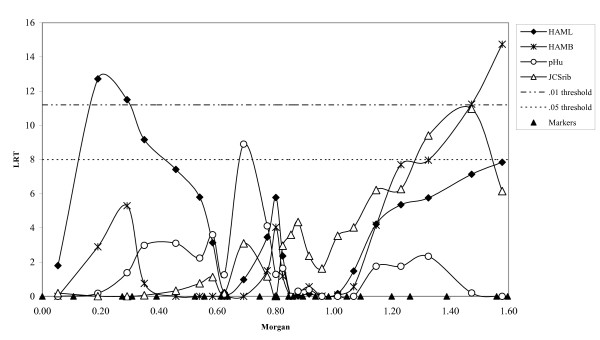
**LRT profiles for meat quality traits with QTL**. Thresholds are corrected for multiple testing and averaged over traits; triangles on the X-axes indicate the location of the markers

**Figure 2:**
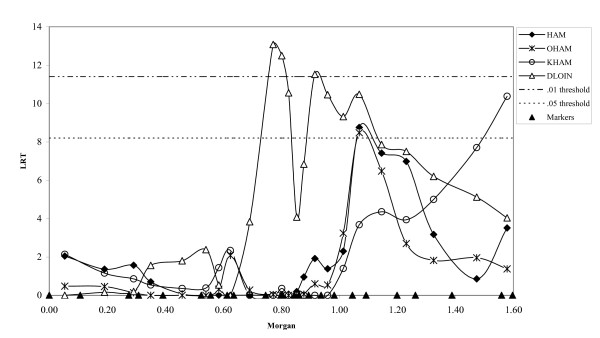
**LRT profiles for carcass quality traits with QTL**. Thresholds are corrected for multiple testing and averaged over traits; triangles on the X-axes indicate the location of the markers

Results are shown for the model applying a single variance component as well as for the two-component model, *i.e*. allowing for different variances among paternal and among maternal haplotypes. The LRT statistics and position of the QTL were very similar for both models. LRT-profiles for meat quality traits with significant QTL are shown in Figure 1 using LRT-values from the two-component model. In Figure 2 similar profiles are shown for carcass quality traits. Applying an analysis using linkage information only (LA-only) showed fewer and less significant QTL (Table 2). Especially for ham-related traits linkage disequilibrium information seems to be of added value.

### Colour

A significant QTL was observed for HAML. The estimated location differed slightly for the two models: 17 cM for the one-component model and 26 cM for the two-component model. Malek *et al*. [11] also found a QTL for this trait on SSC2 but they were located at 72 and 116 cM. (The locations of QTL from other studies are taken from the pigQTLdb [7] where all the map distances are converted to the USDA-MARC map). For HAMB no QTL have previously been reported on SSC2. The JCSrib QTL is in accordance with the QTL for a similar subjective colour score observed by Malek *et al*. [11] although their score was on the cut surface of the loin instead of the side-rib-view. The QTL for HAMB and JCSrib were found at almost the same position, which might indicate that it is the same QTL affecting both traits.

### pH

The QTL for pHu on SSC2 was observed between markers Sw1686 and Sw2167 (65 cM). Lee *et al*. [37] observed two QTL for pHu on SCC2 at 42 and 64 cM in a F2-cross between Meishan and Pietrain. Su *et al*. [38] observed a QTL for pHu at 67 cM. The ultimate pH is usually a good predictor of water holding capacity. Malek *et al*. [11] showed that two QTL are segregating for this trait on SSC2 (around 75 and 114 cM). However, in this study no significant QTL were found for drip or purge.

### Carcass traits

Figure 2 suggests that more than one QTL on SSC2 affect the amount of loin muscle (DLOIN). The most significant QTL for DLOIN at 73 cM on SSC2 has never been reported. Several studies have reported a QTL involving amount of loin at the beginning of SSC2, which is most likely associated with the *IGF2*-gene [22,39,40,37,17]. However, Varona *et al*. [41] and also Lee *et al*. [37] have reported a QTL for loin depth and percentage lean cuts around 65 cM.

Total kg of ham (HAM) as well as part of this ham (OHAM) showed a significant QTL at 103 cM, but the significant QTL for knuckle ham (KHAM) was situated at the end of SSC2. Duthie *et al*. [17] have detected a QTL for ham weight on SCC2 at 15 cM like Vidal *et al*. [14] but this latter study does not give the position.

### Variance components

In Table 3, the proportion of total variance due to polygenic (h^2^), litter (c^2^) and QTL (v^2^) as well as the residual and total variance are given for traits mentioned in Table 2 at the evaluation point where the LRT for the QTL was at its maximum. Given the hybrid origin of the population used in this study, *i.e*. a single strain sire line was crossed with a 3-way cross sow, the two-component model is probably more appropriate than the one-component model because in the two-component model the segregation of the paternal and maternal haplotypes are modelled as independent effects. This is illustrated in Table 3 where contribution of paternal and maternal components is given, *i.e*. v^2^_s _and v^2^_d_.

**Table 3:** Total and residual variance and percentage of variance associated with polygenic, litter and QTL effect (h^2^, c^2 ^and v^2^) for the significant traits using LDLA analysis

		Mendelian model(1)				Two component model(2)				
Trait	total variance	residual variance	h^2^	c^2^	v^2^	residual variance	h^2^	c^2^	v^2^_s_^a^	v^2^_d_
HAML	20.20	17.04	0.01	0.05	0.10	16.75	0.02	0.02	0.04	0.18
HAMB	3.061	2.758	0.03	0.04	0.04	2.782	0.02	0.04	0.06	0.00
pHu	0.018	0.014	0.02	0.14	0.06	0.014	0.02	0.13	0.06	0.05
JCSrib	0.179	0.145	0.07	0.05	0.07	0.143	0.07	0.04	0.05	0.10
										
HAM	0.152	0.118	0.06	0.12	0.04	0.118	0.08	0.11	0.01	0.07
OHAM	0.252	0.199	0.03	0.13	0.05	0.199	0.04	0.12	0.04	0.06
KHAM	0.011	0.008	0.12	0.09	0.04	0.008	0.12	0.09	0.03	0.03
DLOIN	0.066	0.044	0.13	0.16	0.05	0.044	0.13	0.15	0.05	0.04

^a ^for the two-component model, QTL variance was split in paternal and maternal components

In general, proportions of variance due to polygenic and litter effects are in close agreement with van Wijk *et al*. [29] in which data was analysed before marker data was available, *i.e*. they applied a model without QTL effects. The biggest disagreement was observed when comparing the h^2 ^estimates for pHu. The h^2 ^for pHu dropped from 0.11 to 0.02. In both models, the QTL variance (v^2^) is relatively high indicating that the genetic variance has shifted from polygenic to QTL variance. This might be the result of the specific data analysed. Since it is unlikely that a single QTL explains most of the genetic variance, the QTL variance is most likely overestimated.

Different variance components for sire and dam haplotypes for HAMB and HAM indicate that the underlying QTL are not segregating in dam and sires, respectively. Preferably a two-component model should be applied for crossbred data where different QTL alleles could be segregating in different populations involved in the hybrid offspring.

### LDLA

In this study, linkage disequilibrium (LD) information was included when calculating the IBD matrices. However, it is not clear how IBD due to LD should be calculated for crossbred populations. The theory developed by Meuwissen and Goddard [33] assumes a single population 100 generations ago, which is not very likely for very different pig breeds. Uleberg *et al*. [42] have applied an IBD-value of zero due to LD between base-haplotypes of different breeds. Given that all pigs originate from a domesticated wild boar population this seems to be too extreme because haplotypes could be identical by descent due to the single origin. Biodiversity studies, *e.g*. Eding and Meuwissen [43], which provide estimates of genetic distance among breeds, could be used to determine IBD within and between breeds simultaneously.

Compared to Meuwissen *et al*. [44] and Olsen *et al*. [45] the LRT-profiles (Figures 1 and 2) are less peaked. This might due to the lower marker density used in this study or to the use of cross bred data instead of single population data in the other studies, which have a positive effect on linkage disequilibrium information because IBD among founder haplotypes can be better estimated. In particular, the linkage disequilibrium information decreases the width of the peaks because it takes historic recombination into account [44].

## Conclusion

QTL affecting meat and carcass quality were found on SSC2 in this large, commercially produced population. QTL-effects were significant even after correction for multiple testing. The variance component method to detect QTL made it possible to detect QTL segregating in the paternal line (*e.g*. HAMB), the maternal lines (*e.g*. Ham) or in both (*e.g*. pHu). Combining association and linkage information among haplotypes slightly improved the significance of the QTL compared to an analysis using linkage information only.

## Competing interests

The authors declare that they have no competing interests.

## Authors' contributions

RvW and EK organized the phenotypes and performed preliminary analysis. BD and TvK created the genotypes. Statistical analyses were done by RvW and HH. HH and HB wrote the article and supervised the project.
